# Pulmonary Nodular Lymphoid Hyperplasia Presenting With a Reversed Halo Sign

**DOI:** 10.1002/rcr2.70542

**Published:** 2026-03-09

**Authors:** Takafumi Yamano, Shohei Yano, Yuki Yoshida, Moon Hee Hwang

**Affiliations:** ^1^ Department of Respiratory Medicine Osaka Red Cross Hospital Osaka Japan

**Keywords:** ground‐glass nodule, pulmonary nodular lymphoid hyperplasia, reversed halo sign

## Abstract

The reversed halo sign is observed in a broad spectrum of lung diseases, most commonly organising pneumonia, but it has not been reported in pulmonary nodular lymphoid hyperplasia. Herein, we describe the first case of pulmonary nodular lymphoid hyperplasia presenting with a reversed halo sign.

## Graphical abstract

We report a case of pulmonary nodular lymphoid hyperplasia (PNLH) presenting with a reversed halo sign on chest computed tomography. Video‐assisted thoracoscopic wedge resection confirmed a polyclonal reactive lymphoid proliferation without an increase in IgG4‐positive cells. This case highlights that PNLH should be considered in the differential diagnosis of the reversed halo sign and that histologic confirmation is essential.

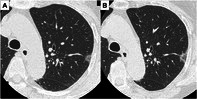

A 76‐year‐old woman, followed up for a left upper lobe ground‐glass nodule detected 7 years earlier, presented with interval enlargement following chest radiography (Figure 1). Chest computed tomography revealed an enlarged ground‐glass nodule with a reversed halo sign (Figure 2). A video‐assisted thoracoscopic wedge resection was performed. Histological analysis revealed clustered reactive lymphoid follicles with perifollicular plasmacytosis. IgG4‐positive cells were not increased (IgG4/IgG < 1%; 0–1/HPF). The κ/λ ratio was 2–3:1, indicating polyclonality and supporting a diagnosis of pulmonary nodular lymphoid hyperplasia (Figure 3). At 9 months follow‐up, there was no recurrence. The reversed halo sign, classically associated with organising pneumonia, also occurs in infections, pulmonary infarction, and granulomatous disease [1]. Pulmonary nodular lymphoid hyperplasia is a rare reactive lymphoproliferative lesion that usually presents as solitary or multiple pulmonary nodules. Pulmonary nodular lymphoid hyperplasia requires distinction from IgG4‐related disease and mucosa‐associated lymphoid tissue lymphoma [2]; immunostaining in our case excluded both. A reversed halo sign, not previously described in patients with pulmonary nodular lymphoid hyperplasia, could be explained by intralesional heterogeneity, with relatively lower central cellularity than peripheral cellularity. The differential diagnosis of reversed halo sign should include pulmonary nodular lymphoid hyperplasia, and tissue diagnosis is essential.

**FIGURE 1:**
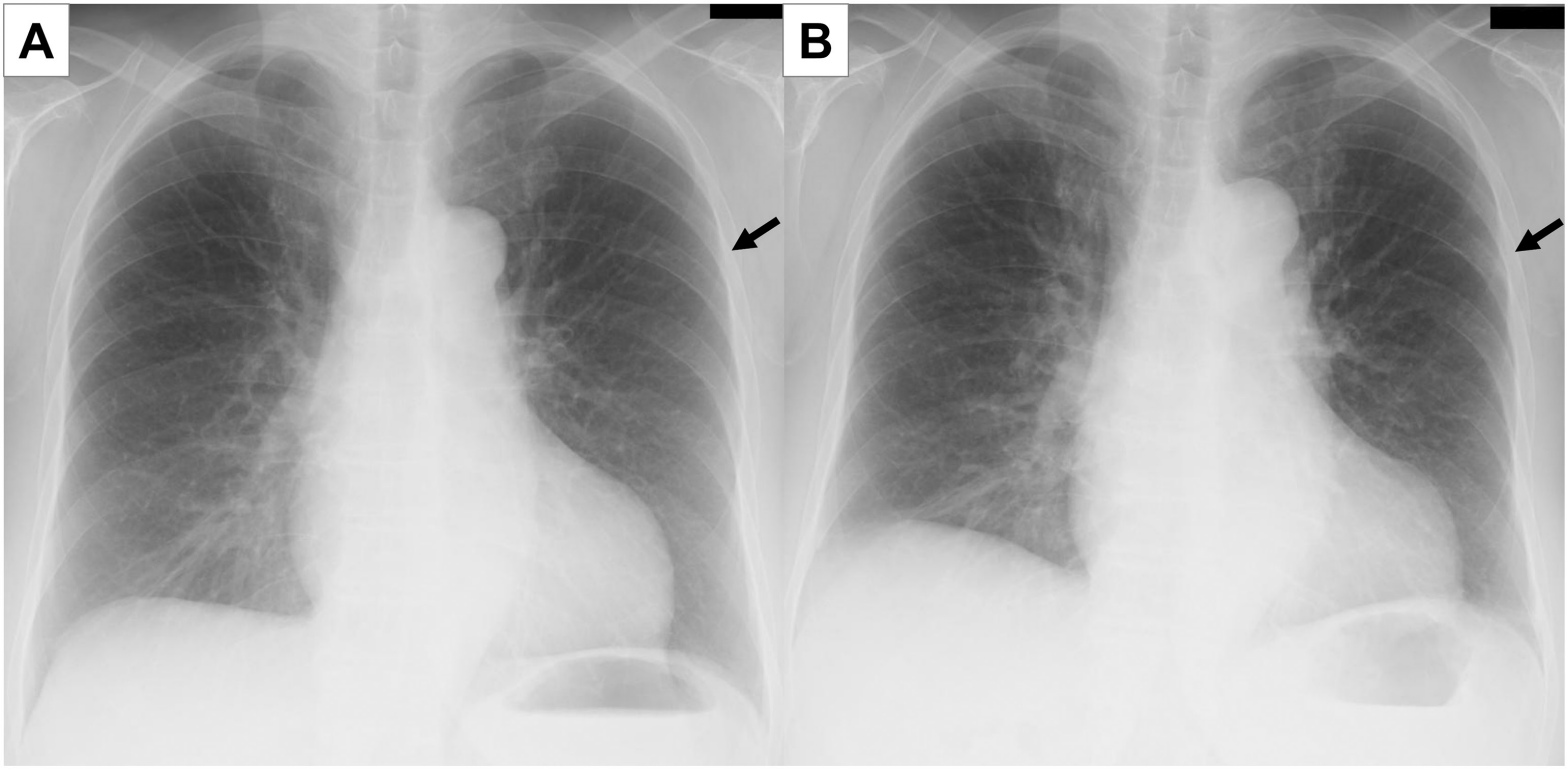
Serial chest radiographs showing interval growth of a left upper‐lung ground‐glass nodule. (A) Seven years prior to presentation, a ground‐glass nodule is observed in the lateral left upper lung field (black arrow). (B) At presentation, the ground‐glass nodule has enlarged (black arrow).

**FIGURE 2:**
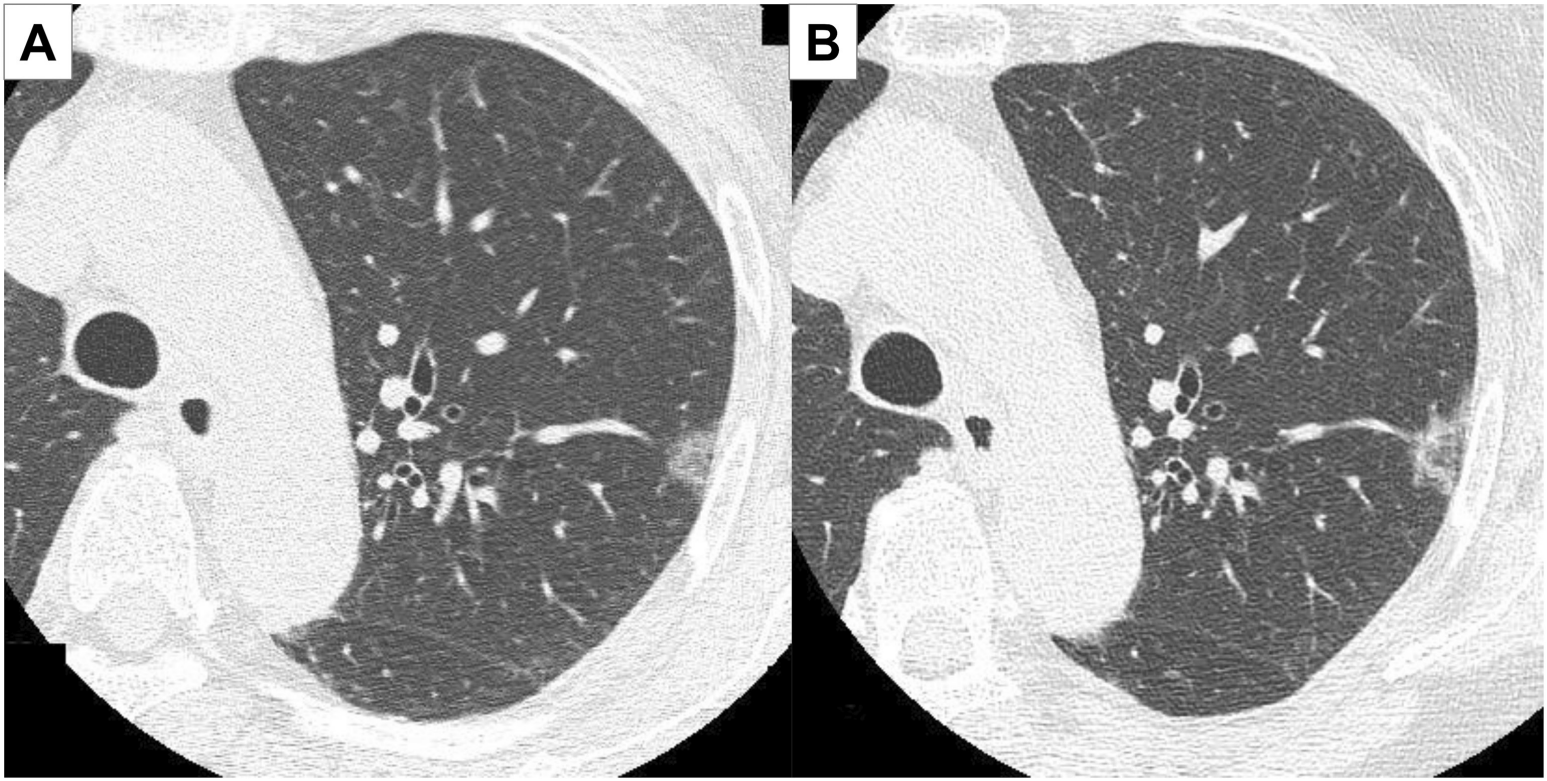
Serial chest computed tomography images showing interval growth of a subpleural ground‐glass nodule with development of the reversed halo sign. (A) Seven years prior to presentation, a subpleural ground‐glass nodule is observed in the left upper lobe. (B) At presentation, the legion is enlarged and exhibits the reversed halo sign.

**FIGURE 3:**
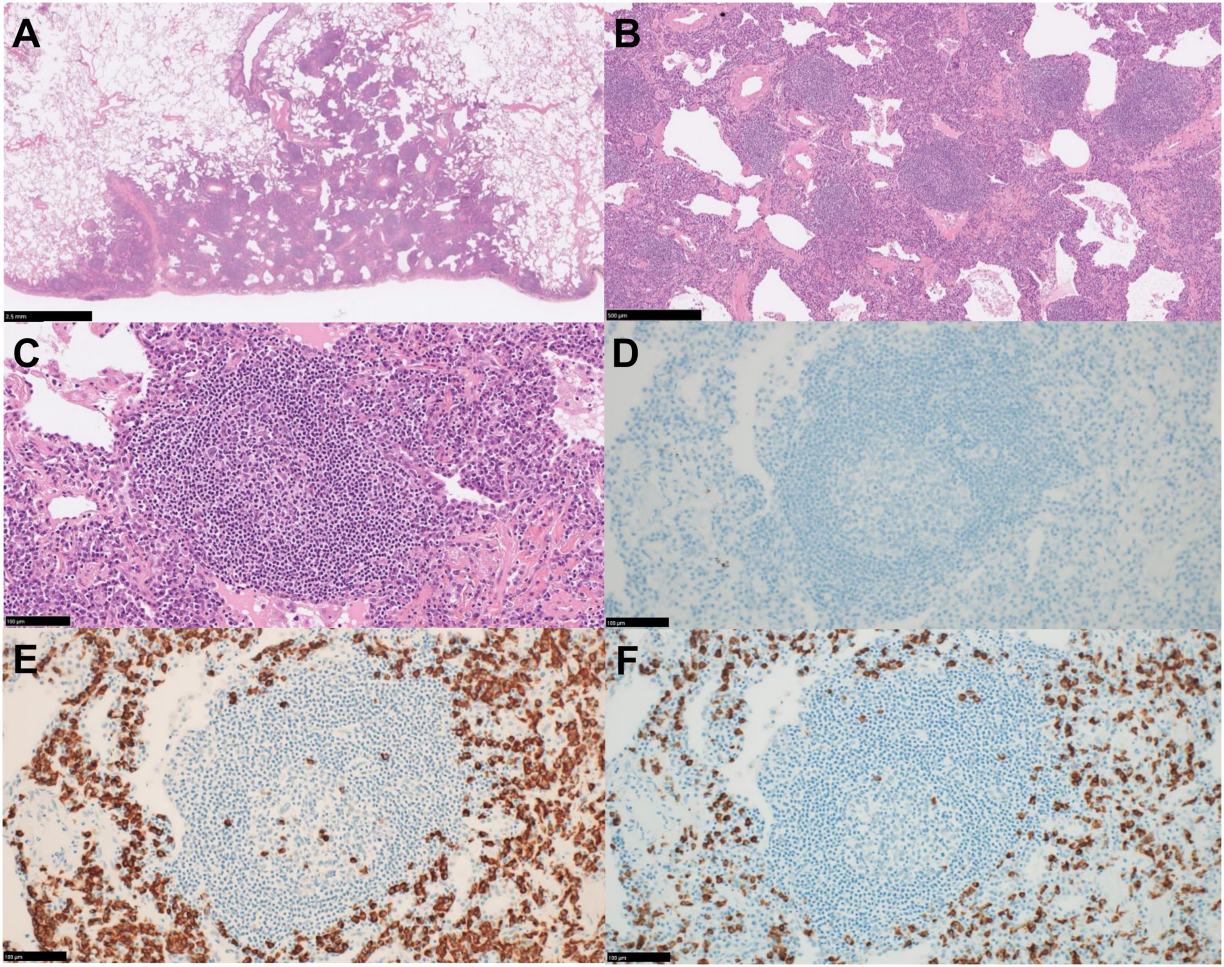
Histopathological features of pulmonary nodular lymphoid hyperplasia in a video‐assisted thoracoscopic surgery specimen. (A) Low‐power view shows a well‐circumscribed nodular lymphoid lesion replacing the adjacent lung parenchyma. (B) Medium‐power view demonstrates numerous reactive lymphoid follicles with prominent germinal centres. (C) Higher‐power view highlights a reactive germinal centre with surrounding small lymphocytes. (D) IgG4 immunostaining shows no significant increase in IgG4‐positive plasma cells. (E, F) Immunostaining for immunoglobulin light chains demonstrates a polytypic plasma cell population without convincing light‐chain restriction (κ in E and λ in F), supporting polyclonality. Haematoxylin and eosin staining (A–C) and immunohistochemistry (D–F). Original magnification: ×1 (A), ×5 (B), ×20 (C–F). Scale bars: 2.5 mm (A), 500 μm (B), 100 μm (C–F).

## Author Contributions

Takafumi Yamano drafted the manuscript and collected the data. Shohei Yano, Yuki Yoshida, and Moon Hee Hwang reviewed and edited the manuscript. All authors approved the final version of the manuscript.

## Funding

The authors have nothing to report.

## Ethics Statement

This case report was approved by the Ethics Committee of the Osaka Red Cross Hospital (approval number: J‐0779). This study was conducted in accordance with the principles of the Declaration of Helsinki.

## Consent

The authors declare that written informed patient consent was obtained for the publication of this manuscript and the accompanying images, and attest that the form used to obtain consent from the patient complies with the journal requirements, as outlined in the author guidelines.

## Conflicts of Interest

The authors declare no conflicts of interest.

## Data Availability

The data that support the findings of this study are available on request from the corresponding author. The data are not publicly available due to privacy or ethical restrictions.

## References

[1] V. N. Maturu and R. Agarwal , “Reversed Halo Sign: A Systematic Review,” Respiratory Care 59, no. 9 (2014): 1440–1449, 10.4187/respcare.03020. 24782557

[2] M. Yell and F. G. Rosado , “Pulmonary Nodular Lymphoid Hyperplasia,” Archives of Pathology & Laboratory Medicine 143, no. 9 (2019): 1149–1153, 10.5858/arpa.2018-0188-RS. 30720334

